# SARS-CoV-2 drives JAK1/2-dependent local complement hyperactivation

**DOI:** 10.1126/sciimmunol.abg0833

**Published:** 2021-04-07

**Authors:** Bingyu Yan, Tilo Freiwald, Daniel Chauss, Luopin Wang, Erin West, Carmen Mirabelli, Charles J Zhang, Eva-Maria Nichols, Nazish Malik, Richard Gregory, Marcus Bantscheff, Sonja Ghidelli-Disse, Martin Kolev, Tristan Frum, Jason R Spence, Jonathan Z. Sexton, Konstantinos D. Alysandratos, Darrell N. Kotton, Stefania Pittaluga, Jack Bibby, Nathalie Niyonzima, Matthew R Olson, Shahram Kordasti, Didier Portilla, Christiane E Wobus, Arian Laurence, Michail S Lionakis, Claudia Kemper, Behdad Afzali, Majid Kazemian

**Affiliations:** 1 Department of Biochemistry, Purdue University, West Lafayette, IN, USA.; 2 Immunoregulation Section, Kidney Diseases Branch, National Institute of Diabetes and Digestive and Kidney Diseases (NIDDK), NIH, Bethesda, MD, USA.; 3 Complement and Inflammation Research Section (CIRS), National Heart, Lung, and Blood Institute (NHLBI), National Institutes of Health (NIH), Bethesda, MD, USA.; 4 Department of Nephrology, University Hospital Frankfurt, Goethe-University, Frankfurt, Germany.; 5 Department of Computer Science, Purdue University, West Lafayette, IN, USA.; 6 Department of Microbiology and Immunology, University of Michigan, Ann Arbor, MI, USA.; 7 Department of Medicinal Chemistry, College of Pharmacy, University of Michigan, Ann Arbor, MI, USA.; 8 GlaxoSmithKline, Stevenage, UK.; 9 Department of Internal Medicine, Gastroenterology, Michigan Medicine at the University of Michigan, Ann Arbor, MI, USA.; 10 Department of Cell and Developmental Biology, University of Michigan, Ann Arbor, MI, USA.; 11 Center for Regenerative Medicine of Boston University and Boston Medical Center, Boston, MA, 1702118, USA.; 12 The Pulmonary Center and Department of Medicine, Boston University School of Medicine, Boston, MA, 02118, USA.; 13 Laboratory of Pathology, Center for Cancer Research, National Cancer Institute (NCI), NIH, Bethesda, MD, USA.; 14 Center of Molecular Inflammation Research (CEMIR), Department of Cancer Research and Molecular Medicine, Norwegian University of Science and Technology (NTNU), 7491 Trondheim, Norway.; 15 Department of Biological Sciences, Purdue University, West Lafayette, IN, USA.; 16 CRUK-KHP Centre, Comprehensive Cancer Centre, Kings College London, London, UK.; 17 Haematology Department, Guys Hospital, London, UK.; 18 Division of Nephrology and the Center for Immunity, Inflammation and Regenerative Medicine, University of Virginia, VA, USA.; 19 Nuffield Department of Medicine, University of Oxford, UK.; 20 Fungal Pathogenesis Section, Laboratory of Clinical Immunology and Microbiology, National Institute of Allergy and Infectious Diseases (NIAID), NIH, Bethesda, MD, USA.; 21 Institute for Systemic Inflammation Research, University of Lbeck, Lbeck, Germany.

## Abstract

Patients with coronavirus disease 2019 (COVID-19) present a wide range of acute clinical manifestations affecting the lungs, liver, kidneys and gut. Angiotensin converting enzyme (ACE) 2, the best-characterized entry receptor for the disease-causing virus SARS-CoV-2, is highly expressed in the aforementioned tissues. However, the pathways that underlie the disease are still poorly understood. Here, we unexpectedly found that the complement system was one of the intracellular pathways most highly induced by SARS-CoV-2 infection in lung epithelial cells. Infection of respiratory epithelial cells with SARS-CoV-2 generated activated complement component C3a and could be blocked by a cell-permeable inhibitor of complement factor B (CFBi), indicating the presence of an inducible cell-intrinsic C3 convertase in respiratory epithelial cells. Within cells of the bronchoalveolar lavage of patients, distinct signatures of complement activation in myeloid, lymphoid and epithelial cells tracked with disease severity. Genes induced by SARS-CoV-2 and the drugs that could normalize these genes both implicated the interferon-JAK1/2-STAT1 signaling system and NF-B as the main drivers of their expression. Ruxolitinib, a JAK1/2 inhibitor, normalized interferon signature genes and all complement gene transcripts induced by SARS-CoV-2 in lung epithelial cell lines, but did not affect NF-B-regulated genes. Ruxolitinib, alone or in combination with the antiviral remdesivir, inhibited C3a protein produced by infected cells. Together, we postulate that combination therapy with JAK inhibitors and drugs that normalize NF-B-signaling could potentially have clinical application for severe COVID-19.

## Teaser

SARS-CoV-2 induces JAK-STAT1dependent complement C3 transcription and processing by a cell-intrinsic alternative pathway convertase.

## INTRODUCTION

Coronavirus disease 2019 (COVID)-19, a viral pneumonia caused by a beta coronavirus named severe acute respiratory syndrome coronavirus (SARS-CoV)-2, is now a pandemic. Patients with COVID-19 present variable clinical symptoms, ranging from a mild upper respiratory tract illness to a significant disease with severe and life-threatening complications, characterized by combinations of acute respiratory distress syndrome, coagulopathy, vasculitis, kidney, liver and gastrointestinal injury (*1*). Survivors, and those with milder presentations, may suffer from loss of normal tissue function due to persistent inflammation and/or fibrosis. (*2*, *3*). The pathogenesis of COVID-19 and the causes of its variable severity are poorly understood, thus a better mechanistic understanding of the disease will help identify at-risk patients and allow for the development and refinement of much-needed treatments.

The complement system is an evolutionarily conserved component of innate immunity, required for pathogen recognition and removal (*4*). The key components are complement (C)3 and C5, which circulate in their pro-enzyme forms in blood and interstitial fluids. C3 is activated through the classical (antibody signal), lectin (pattern recognition signal) and/or alternative (altered-self and tick-over) pathways into bio-active C3a and C3b via cleavage by an enzyme complex called C3 convertase. Complement factor B (CFB) is a key component of the alternative pathway C3 convertase. C3b generation triggers subsequent activation of C5 into C5a and C5b, with the latter seeding the formation of the lytic membrane attack complex (MAC) on pathogens or target cells. C3a and C5a are anaphylatoxins and induce a general inflammatory reaction by binding to their respective receptors, C3a receptor (C3aR) and C5aR1 expressed on immune cell. C3b binds its canonical receptor, CD46, which is expressed on nucleated cells and acts as both a complement regulator and a driver of T helper 1 differentiation in CD4^+^ T cells (*5*, *6*). Although the traditional view of complement is as a hepatocyte-derived and serum-effective system, the complement system is also expressed and biologically active within cells (*7*).

Patients with severe COVID-19 have high circulating levels of terminal activation fragments of complement (C5a and sC5b-9) (*8*,*10*), which correlate to disease severity (*8*). Single nucleotide variants in two complement regulators, decay accelerating factor (CD55) and complement factor H, are risk factors for morbidity and mortality from SARS-CoV-2 (*11*). This is concordant with a recent report, which shows that serum C3 hyperactivation is an independent risk factor for in-hospital mortality (*12*). Despite these reports, the mechanisms behind the overactivation and conversion of the normally protective complement system into a harmful component of COVID-19 are currently unclear.

Here, we examined the transcriptomes of respiratory epithelial cells infected with SARS-CoV-2 and found that the complement system was one of the intracellular pathways most highly induced in response to infection. C3 protein was processed to active fragments by expression of an inducible alternative pathway convertase (CFB) and that was normalized by a cell-permeable inhibitor of CFB. Interferon signaling via the JAK1/2STAT1 pathway was principally responsible for transcription of complement genes in this setting and ruxolitinib, a JAK1 inhibitor, alone or in combination with remdesivir, an anti-viral agent, normalized this transcriptional response and production of processed C3 fragments from infected cells.

## RESULTS

### SARS-CoV-2 infection activated complement transcription in lung epithelial cells

**Fig. 1:**
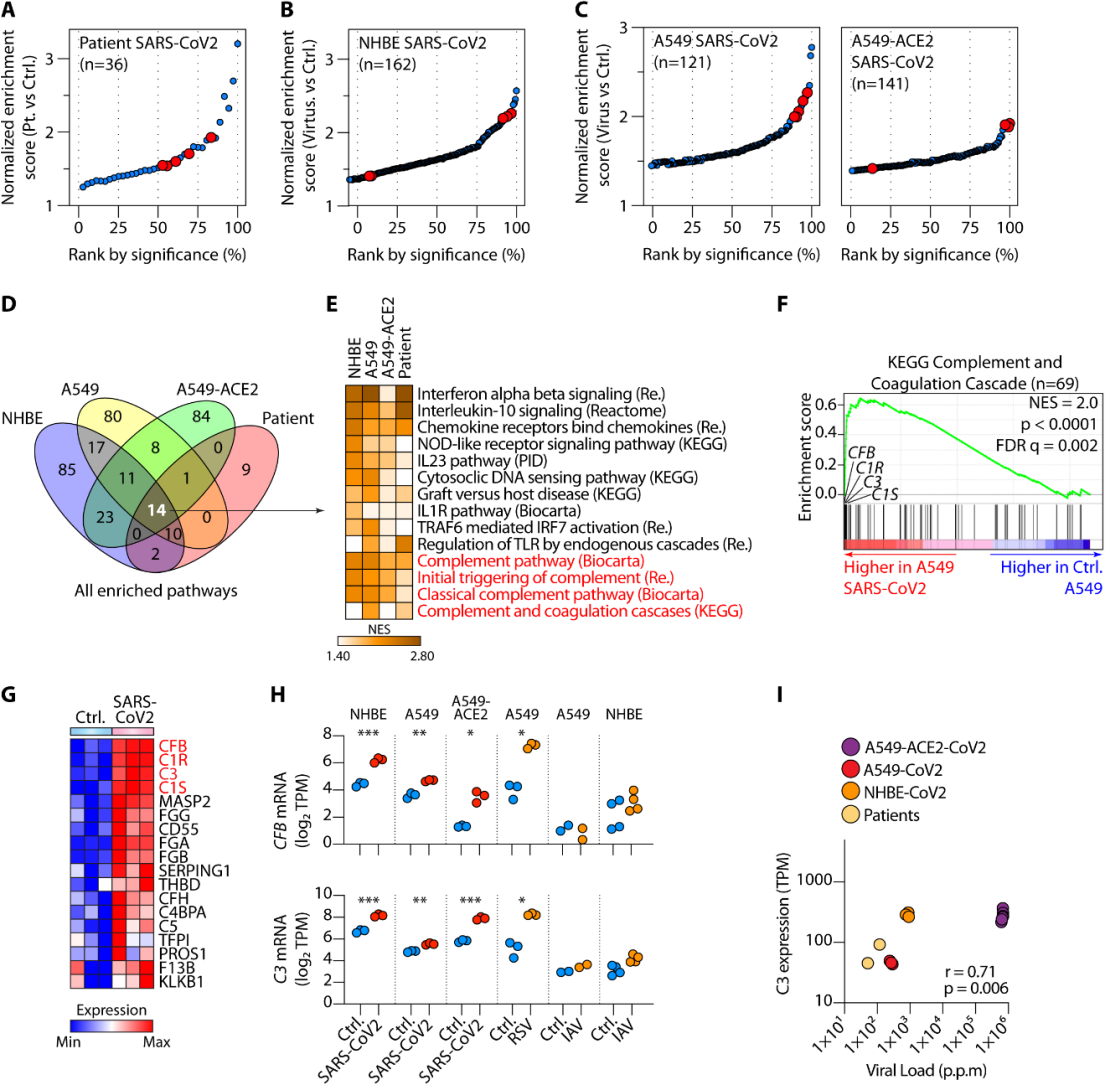
**SARS-CoV-2 infection activated complement transcription in lung epithelial cells. (A-B)** Significantly enriched pathways by gene set enrichment analysis (GSEA) comparing transcriptomes of lung samples from SARS-CoV-2 infected patients (n=2) versus uninfected controls (**A**) and similar GSEA analyses on normal human bronchial epithelial (NHBE) cells infected in vitro, or not, with SARS-CoV-2 (n=3) (**B**). (**C**) GSEA of A549 cells transduced with ACE2 (A549-ACE2) or not (A549), comparing cells infected with SARS-CoV-2 versus control cells (n=3 or 4). Pathways in **A-C** were ranked by significance (false-discovery rate FDR q-values), with complement pathways highlighted in red. Only enriched pathways with FDR <0.25 are shown. **(D-E)** Comparison of all pathways significantly induced (FDR q-value < 0.25) by SARS-CoV-2 in patients (**A**), NHBE cells (**B**), A549 and A549-ACE2 cells (**C**), indicating 14 shared enriched pathways (**D**) and their normalized enrichment score (NES) displayed as a heatmap, with complement pathways highlighted in red (**E**). (**F-G**) Representative GSEA plot for one of the complement pathways in (**E**) and expression of the leading-edge genes from this pathway, with *C3*, *C1R*, *C1S* and *CFB* highlighted in red (**G**). (**H**) Expression of *CFB* (upper panel) and *C3* (lower panel) mRNA in control (Ctrl.) versus SARS-CoV-2-infected cells. (**I**) Spearman correlation between *C3* mRNA expression and SARS-CoV-2 viral load across virus bearing samples in **Fig. 1A-H**. p.p.m: parts per million mapped reads. Data have been sourced from GSE147507. *p <0.05, ** p <0.01, ***p < 0.001, by ANOVA.

To gain insights into the pathophysiologic mechanisms of COVID-19, we sourced bulk RNA-seq data from lung tissues of two patients with SARS-CoV-2 infection and uninfected controls (**Table S1A**) (*13*). We compared the transcriptomes of patients to controls using gene set enrichment analysis (GSEA) (*14*) and found 36 canonical pathways curated by the Molecular Signatures Database (MSigDB) to be induced in patients compared to controls (**Fig. 1A** and **Table S1B**). Five of the 36 (14%) enriched pathways were annotated as complement pathways. Traditionally, complement is considered a mostly hepatocyte-derived and serum-effective system (*4*). Thus, the dominance of the SARS-CoV-2-induced lung cell-intrinsic complement signature was unexpected.

Since the patient lung biopsy samples contained a mixed population of lung cells, we next defined the cellular source of the complement signature in the affected lungs. To this end, we examined the transcriptomes of primary human bronchial epithelial (NHBE) cells infected in vitro with SARS-CoV-2, which again identified several complement pathways as highly enriched in infected cells. In fact, hierarchical classification of enriched pathways by significance (FDR q-value) showed that complement pathways were among the most highly enriched of all pathways following SARS-CoV-2 infection (**Fig. 1B**). One of the cell types infected by SARS-CoV-2 are type II pneumocytes, which are high expressors of ACE2, the best characterized entry receptor for the virus (*15*). We, therefore, examined the transcriptomes of A549 cells, which have properties of type II human pneumocytes (*16*, *17*), infected with SARS-CoV-2, and A549 cells first transduced to express high levels of ACE2. Complement pathways were among the most highly enriched, one of which was the most significantly induced pathway in ACE2-transduced A549 cells (**Fig. 1C**) (*13*). This response was much more pronounced for SARS-CoV-2, as analysis of RNA-seq of influenza A-infected NHBE or influenza A- or RSV-infected A549 cells did not induce such dramatic pathway enrichment (**Figs. S1A-B** and **Table S1B**), even though viral loads in infected samples were comparable (**Fig. S1C**).

To further pinpoint common modes of function, we compared all SARS-CoV-2-induced pathways among the four sample types infected with this virus: patient lung biopsies, NHBE, A549 and A549-ACE2 cells. Of the 14 pathways that were significantly induced by SARS-CoV-2 in all sample types, four of them were complement related (**Figs. 1D-E**). The other shared pathways predominantly included antiviral responses, especially type I interferons (IFNs) (**Fig. 1E**). Taking the KEGG complement and coagulation pathway, we noted that genes whose transcription was most highly induced by SARS-CoV-2 were encoding components of the C1 proteases C1R and C1S, complement factor B (CFB) and complement C3 (**Figs. 1F-H** and **Fig. S1D**). C1 proteases are initiators of the classical pathway of complement activation, CFB is essential for the formation of alternative pathway C3 convertase (C3bBb) that activates C3 and C3 is the fundamental rate-limiting substrate for both (*4*). These data were supported by apparent dose-dependency between SARS-CoV-2 viral loads in infected samples and C3 expression (**Fig. 1I**). To further test our conclusions, we analyzed transcriptomes of human bronchial organoids (hBOs) infected with SARS-CoV-2. These also showed that genes more highly expressed in infected hBOs were enriched in complement genes, including *C3* and *CFB* (**Figs. S2A-B**). A recent single cell RNAseq study of human bronchial epithelial cells (HBECs) infected, or not, with SARS-CoV-2 in air-liquid interface cultures has identified eight cell types, four of which are actively infected by the virus (basal cells, basal/club intermediate cells, club cells and ciliated cells) (*18*). Using these data, we carried out GSEA on the ranked list of DEGs, provided by the authors, in each cell type and looked for the enrichment of hallmark gene sets curated by MSigDB. We found that hallmark complement pathway genes were enriched in only the four cell types infected with SARS-CoV-2 but in none of the uninfected cells (**Fig. S2C**). The complement pathway was particularly induced in club cells (*19*) (**Fig. S2C**). *C3* was one of the most significantly enriched genes within the leading edge (**Figs. S2D-E**). Proteomic analysis of mass spectrometry data from A549-ACE2 cells infected, or not, with SARS-CoV-2 confirmed the increased production of C3 protein following infection (**Fig. S3**), consistent with the observed transcriptomic data. Collectively, these data suggested that the complement system was one of the top pathways activated by SARS-CoV-2 in lung epithelial cells.

### C3 protein was processed to active forms in SARS-CoV-2-infected cells

**Fig. 2:**
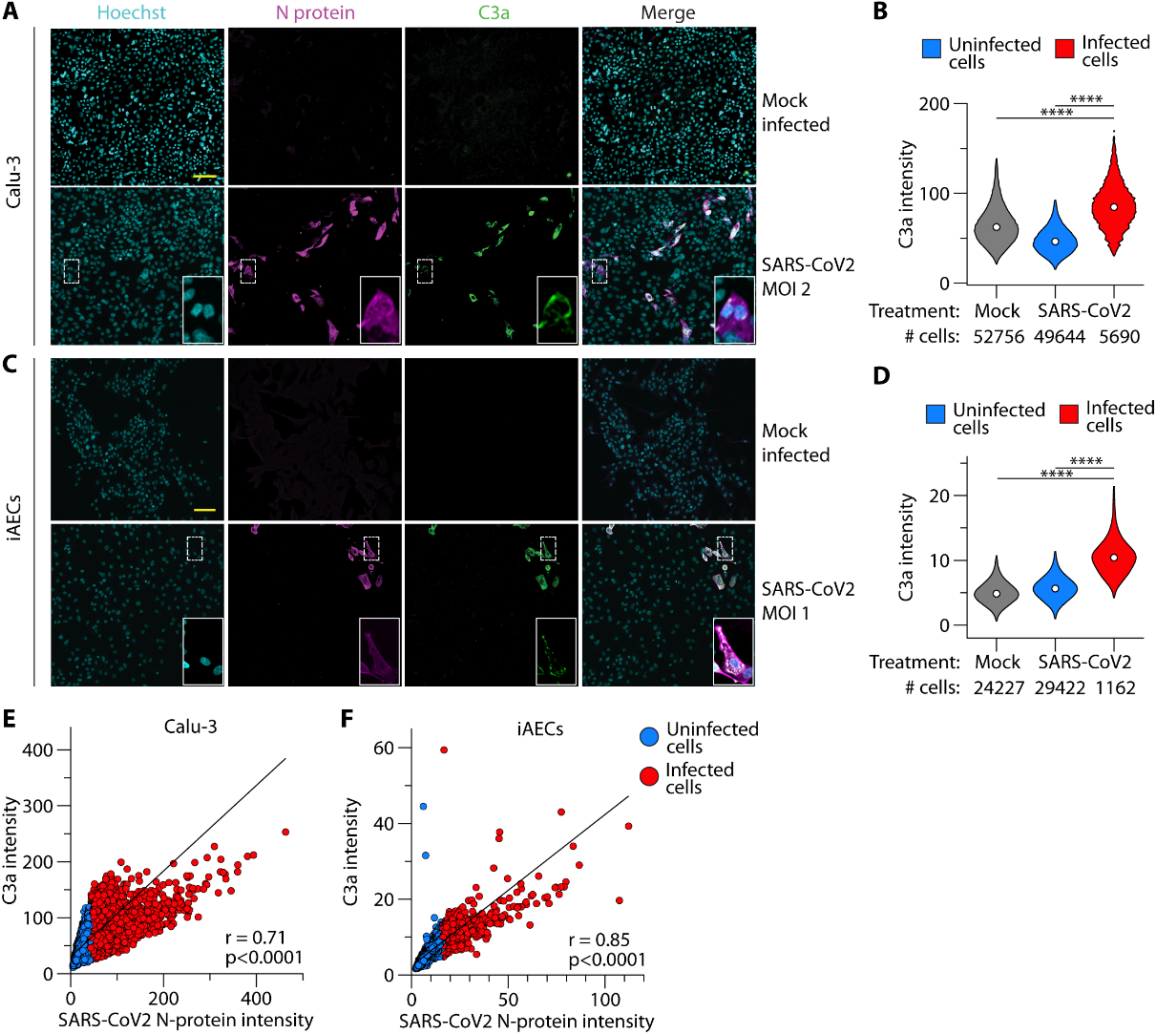
SARS-CoV-2 infection generated C3a protein in lung epithelial cells. (**A-D**) Confocal images (**A** and **C**) and quantification (**B** and **D**) from *n*=2 independent experiments showing expression of C3a and SARS-CoV-2 N-protein in SARS-CoV-2-treated or mock-infected Calu-3 cells (**A** and **B**) or induced pleuripotent stem cell-derived alveolar epithelial type 2 cells (iAEC2s) (**C** and **D**). Scale bars in **A** and **C** indicates 100m. Cell numbers are indicated below each violin and median values denoted by dots in **B** and **D**. (**E-F**) correlation between SARS-CoV-2 N-protein intensity and C3a intensity on a per cell basis in Calu-3 cells (**E**) and iAEC2s (**F**). Indicated are Pearson correlation coefficients and associated p-values. Infected and uninfected cells in (**B-D**) have been distinguished by red and blue fills, respectively. ****p<0.0001 by ANOVA.

C3 is the rate limiting step of distal complement component activation (C5 to C9) and its own processing generates the biologically active fragments C3a and C3b (*4*). C3 is activated through an enzyme complex called C3 convertase (*4*).Viral induction of both *C3* and *CFB* within epithelial cells (**Figs. 1F-H**) suggested the presence of an intracellular C3 convertase capable of processing C3 to its active fragments. To determine whether C3 protein is activated to the C3a fragment in infected cells, we infected Calu-3 cell lines and primary human induced pluripotent stem cell-derived alveolar epithelial type 2 cells (iAEC2s) with SARS-CoV-2 and measured C3a by confocal imaging. In both Calu-3 and iAEC2s we observed minimal C3a in mock-infected cells (**Figs. 2A** **-D**). In cultures treated with SARS-CoV-2, infected cells had significantly increased intracellular C3a compared to both uninfected and mock-infected cells (**Figs. 2A-D**). There was a direct linear correlation between SARS-CoV-2 N-protein expression and C3a levels in both cell types (**Fig. 2E-F**), indicating a relationship between C3 activation in lung epithelial cells and viral load. Collectively, these data showed that respiratory epithelial cells were a source of complement C3 and its active products.

### SARS-CoV-2 infection invoked distinct complement signatures across immune and epithelial cells in patients

**Fig. 3:**
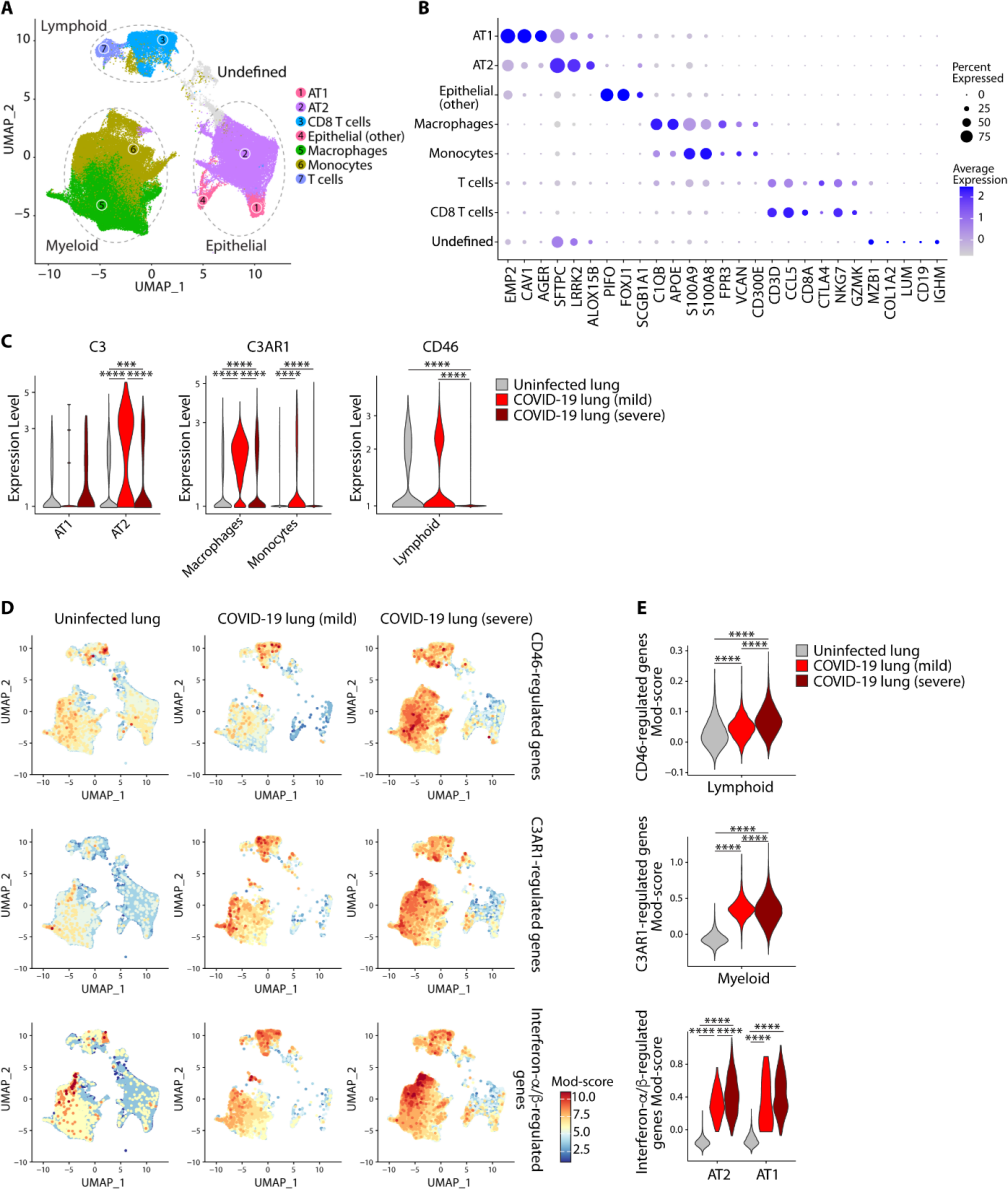
**SARS-CoV-2 infection invoked distinct complement signatures across lymphoid, myeloid and epithelial cells in patients. (A)** UMAP showing 3 major cell types and 7 sub-cell types in uninfected subject lung biopsies (*n*=8) and COVID-19 bronchoalveolar lavage (BAL) specimens from patients with mild (*n* =3) and severe (*n* =3) COVID-19. (**B**) Expression of cell-defining features across all cell types. (**C**) Expression of *C3*, *C3AR1* and *CD46* in select cell types across uninfected, mild and severe COVID-19 samples (see also **Figs. S4A-B** for all cell types). (**D-E**) The UMAP projection (**D**) and module (Mod) score (*54*) (**E**) of CD46-regulated genes (top panel), C3aR1-regulated genes (middle panel) and interferon-/-regulated genes (see **Table S2**). In (**E**) selected cell types are shown. Single cell data are from GSE145926 and GSE122960. ****p<0.0001 by Wilcoxon test.

To obtain insight into the interactions between SARS-CoV-2 and the complement system in vivo, we analyzed publicly available single cell RNA-sequencing data from patients with COVID-19 (*20*). Bronchoalveolar lavage (BAL) samples from patients with mild (n=3) and severe (n=3) COVID-19 were compared with lung biopsy samples from uninfected individuals (n=8). Clustering across all cells revealed three major cell types of myeloid, lymphoid and epithelial origin, with seven apparent sub-cell types (**Figs. 3A-B**). We distinguished alveolar type I (AT1) and type II pneumocytes (AT2) (**Figs. 3A-B**). We found that expression of *C3* was highest in AT2 cells (**Fig. 3C**, **left panel** and **top panels** of **Figs. S4A-B**), which have high expression of ACE2 and are major targets of primary SARS-CoV-2 infection (*15*). Although absolute cellularity was different between patients and uninfected subjects due to differences in tissue source (lung biopsy versus BAL, respectively), this difference had minimal effect on our observations. Expression of *C3* was significantly higher in AT2 cells of patients with COVID-19 than those of uninfected donors (**Fig. 3C**), indicating that coronavirus infection of these cells induces *C3* gene transcription in vivo. In an independent (bulk) RNA-seq dataset of bronchoalveolar fluid cells from (*n*=8) patients with COVID-19 and (*n*=20) uninfected controls, we found similar enrichment of complement genes in cells from patients, including significantly higher expression of *C3* and *CFB* (**Figs. S5A-B**). Likewise, lung samples collected at autopsy from patients with COVID-19 showed a positive linear relationship between SARS-CoV-2 viral loads and *C3* mRNA expression (**Fig. S6**), consistent with the dose-dependency observed between these two parameters on experimental SARS-CoV-2 infection (**Figs. 2E-F**).

The biologically active components of C3, C3a and C3b, bind their cognate receptors C3aR and CD46, respectively, on leukocyte subsets to activate these cells and drive inflammation. As expected, we saw high expression of *C3AR1*, the gene encoding C3aR protein, on myeloid cells (**Fig. 3C** **middle panel** and **middle panels** of **Figs. S4A-B**) and CD46 on lymphoid cells (**Fig. 3C** **right panel** and **lower panels** of **Figs. S4A-B**). To determine whether C3 within lung tissues is biologically active, we looked for the signature of genes regulated by C3aR and CD46. We curated a list of genes regulated by CD46 in lymphoid cells (**Table S2**). Expression of CD46-regulated genes was significantly higher in lung lymphoid cells of patients and was higher in more severe cases (**Figs. 3D-E** **top panels**). In an independent (bulk) RNA-seq dataset of bronchoalveolar fluid cells from patients with COVID-19, we found similar enrichment of CD46-regulated genes in patient cells compared to uninfected controls (**Fig. S7**). Similarly, we compiled a list of genes regulated by C3aR in myeloid cells (**Table S2**). C3aR-regulated genes were significantly more expressed in monocyte/macrophage cells of patients compared to those from uninfected individuals (**Figs. 3D-E** **middle panels**). We also analyzed single cell RNA-seq of circulating immune cells within peripheral blood mononuclear cells (PBMC) of patients and healthy controls. In contrast to what we had seen in the lungs, *C3* was minimally expressed by circulating immune cells and the signatures of CD46 and C3aR activation were absent in patients cells (**Figs. S8A-C**). Collectively, these data indicated that C3 was produced locally in the lungs of COVID patients and processed to active fragments that acted on their cognate receptors to drive inflammation.

### Complement gene transcription in lung epithelial cells was JAK1/2-STAT1dependent

**Fig. 4:**
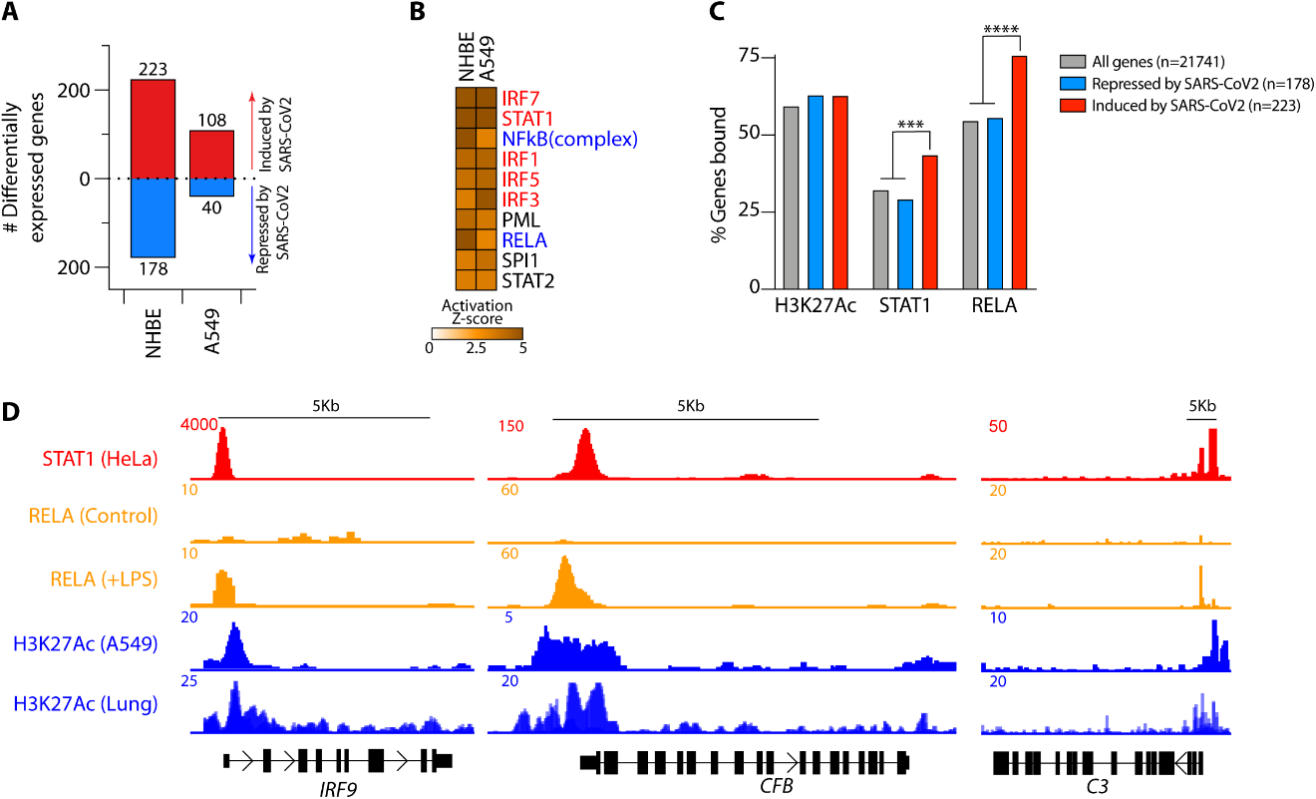
STAT1 and RELA bound to complement genes induced by SARS-CoV-2. (**A**) Numbers of differentially expressed genes in normal primary human bronchial epithelial (NHBE) cells and A549 alveolar cell lines infected with SARS-CoV-2 in comparison with mock infection. (**B**) The top ten Ingenuity Pathway Analysis (IPA) predicted transcription factors (TFs) regulating the SARS-CoV-2-driven transcriptional response in normal human bronchial epithelial (NHBE) cells and human alveolar basal epithelial cell lines (A549). Highlighted in red are TFs transducing interferon-mediated and in blue NF-B-mediated gene transcription. (**C**) Histone 3 lysine 27 acetylation (H3K27Ac) and STAT1 and RELA ChIP-seq binding profiles across SARS-CoV-2-induced and repressed genes. (**D**) STAT1, RELA and H3K27Ac ChIP-seq tracks showing the *IRF9*, *CFB* and *C3* gene loci. Data in **A** are from GSE147507 and in **C-D** have been sourced from ENCODE (H3K27Ac and STAT1) and from GSE132018 (RELA). RELA profiles in **C** are from LPS-treated cells. *** p<0.001; ****p<0.0001 by Fishers exact test.

We next evaluated the if type I IFN responses played a role in complement activation, as IFNs were a common pathway activated by SARS-CoV-2 in respiratory epithelial cells (**Fig. 1E**). We sourced IFN-/ signaling genes from Reactome (R-HSA-909733) (**Table S2**). Genes regulated by type I IFNs were elevated in most cells from patients compared to uninfected controls including in AT1 and AT2 cells (**Figs. 3D-E** **lower panels**). CD46, C3aR and IFN-/ signaling genes appeared to closely track with disease severity in lymphoid, myeloid and pneumocyte (AT1 and AT2) cells, respectively. The correlation between both IFN and C3 in epithelial cells led us to explore the possibility that there may be a causal relationship between the two and mediated by transcription factors driven by IFNs. We assessed the genes differentially regulated by SARS-CoV-2 in primary normal human bronchial epithelial cells and the type II pneumocyte-like (A549) cell line. SARS-CoV-2 induced 223 and 108, and repressed 178 and 40 genes, in NHBE and A549 cells, respectively (**Fig. 4A** and **Table S3A**). We employed ingenuity pathway analysis (IPA) to predict the transcriptional regulators of these genes. Of the top ten TFs predicted, half were IFN-pathway signaling proteins, including STAT1 (**Fig. 4B**), the JAK1/2-induced STAT that transduces signals downstream of the IFN- receptor (*20*). Two of the other core TFs were NF-B family TFs, including RELA (**Fig. 4B**), a major regulator of gene transcription in response to pathogen and inflammatory cytokines (e.g., TNF and IL-1) (*21*). To validate whether STAT1 directly regulated complement genes, we analyzed publicly available ChIP-Seq datasets of STAT1 and histone 3 lysine 27 acetylation (H3K27Ac, a marker of active and open chromatin regions) curated by ENCODE, as well as a RELA ChIP-Seq dataset from GSE132018. Genes regulated by SARS-CoV-2 showed significant enrichment for both STAT1 and RELA binding (**Fig. 4C**). Both TFs bound open chromatin regions (H3K27 acetylated) of genes induced by SARS-CoV-2 in COVID-19 patient lung tissue and in normal human bronchial epithelial cells (NHBE) (**Fig. S9A-B** and **Table S3B**). This indicated that STAT1 and RELA were strongly binding to the promoter regions of *C3*, *CFB*, *C1S*, *C1R*, *IRF9*, *IRF7* and *IL6*, suggesting a potential role in their regulation (**Fig. 4D** and **Fig. S9C**). Together, these data provided strong evidence that genes encoding complement components were regulated by STAT1 and RELA.

### JAK/STAT inhibitors were predicted to normalize SARS-CoV-2-driven complement gene transcription

**Fig. 5:**
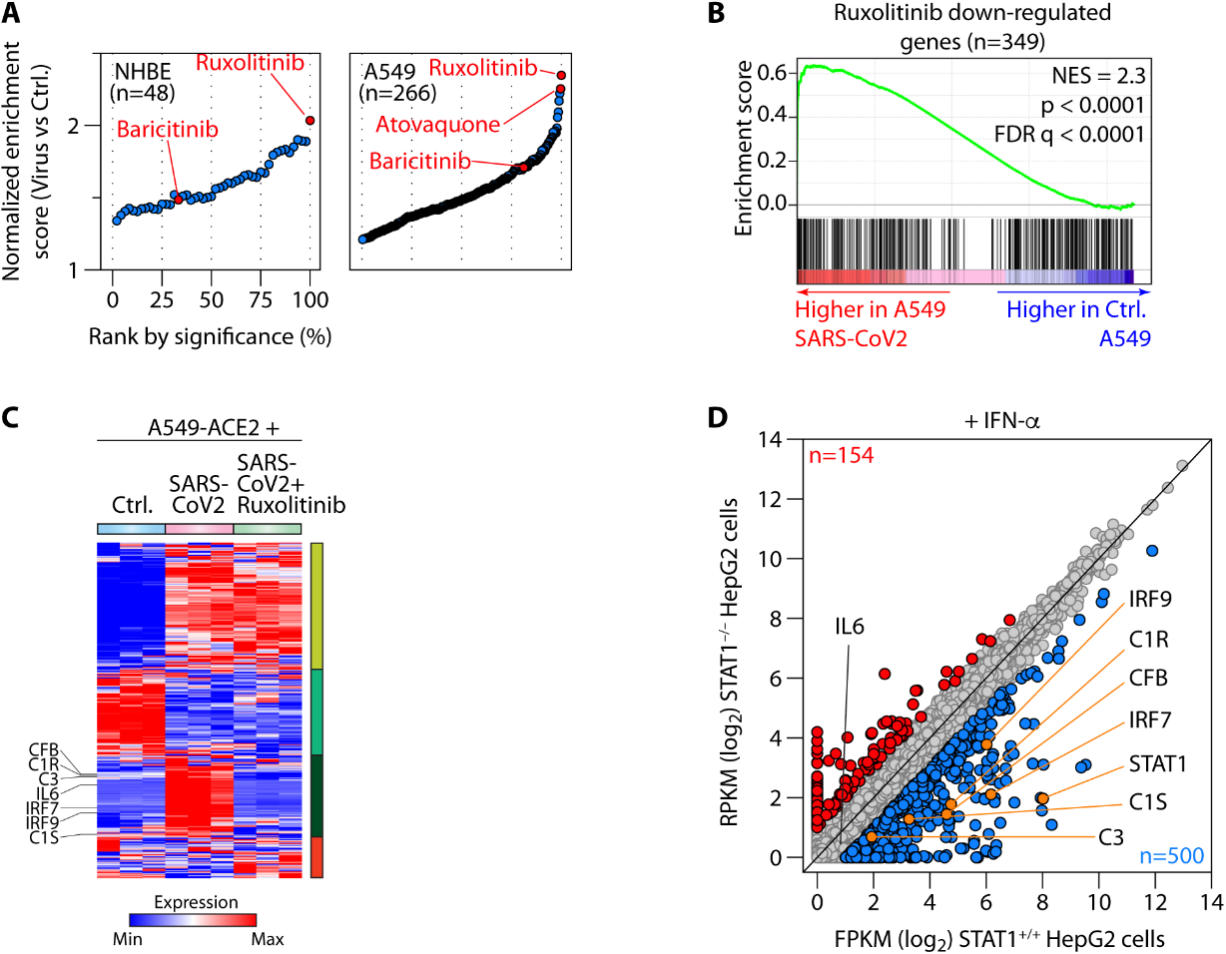
The Janus kinase inhibitor (JAKi) ruxolitinib neutralized SARS-CoV-2 mediated complement transcription. (**A**) Gene set enrichment analysis (GSEA) showing enrichment of genes normalized by pharmaceutical agents in the transcriptomes of control (Ctrl.) or SARS-CoV-2-infected NHBE (left) or A549 (right) cells. Drugs have been ranked by significance (false-discovery rate q-values), with ruxolitinib, baricitinib and atovaquone highlighted in red. (**B**) Representative GSEA plot showing enrichment (higher expression) of ruxolitinib down-regulated genes in SARS-CoV-2-treated cells. (**C**) Heatmap showing expression of genes induced/repressed by SARS-CoV-2 in A549 cells transduced with ACE2 (A549-ACE2) then infected with SARS-CoV-2 in the presence of ruxolitinib or vehicle. Genes are clustered according to their response to SARS-CoV-2 and ruxolitinib. (**D**) Scatter plot comparing the expression of all genes between STAT1 wild-type (*STAT1^+/+^*) and STAT1 knockout (*STAT1^/^*) HepG2 cells after interferon (IFN)- treatment. Differentially expressed genes (Fold change>2) are highlighted in blue (down-regulated in knockout) and red (up-regulated in knockout) and selected key complement and interferon pathway genes highlighted in orange. *IL6* is also marked but not significantly expressed or changed. Transcriptomes are sourced from GSE147507 (**A-C**) (*13*) and GSE98372 (**D**) (*25*).

In parallel, we carried out pharmaceutical drug prediction. To this end, we compared the targets of 1657 curated drugs in the drug signatures database (DSigDB) (*22*) to the genes induced by SARS-CoV-2 infection. In both primary NHBE and A549 cells ruxolitinib, a Janus kinase (JAK)1/2 inhibitor (JAKi) that blocks STAT1 signaling (*23*), was predicted to be the top candidate for normalization of the SARS-CoV-2 gene signature (**Figs. 5A-B** and **Table S4A**), consistent with the enrichment of STAT1 binding in genes regulated by this virus (**Figs. 4C-D**).

We analyzed the effects of ruxolitinib on the SARS-CoV-2-induced transcriptome by comparing RNA-seq from A549 cells transduced to express ACE2, then infected with SARS-CoV-2 in the presence of ruxolitinib or vehicle (**Fig. 5C**). *IL6*, *IRF7* and *IRF9* and all of the complement components we had previously observed, namely *C1R*, *C1S*, *CFB* and *C3*, were almost completely normalized by ruxolitinib (**Fig. 5C** and **Table S4B**).

JAKi can have off-target effects (*24*) and STAT3 can theoretically be activated by the IL-6 produced in response to SARS-CoV-2, thus we confirmed if complement was regulated by STAT1 in a STAT1 deficient cell line. We analyzed publicly available transcriptomes of STAT1 wild-type (*STAT1^+/+^*) and STAT1 knockout (*STAT1^/^*) HepG2 liver cells treated, or not, with IFN- (*25*). Treatment with IFN-a, an archetypal STAT1-activating type I interferon, is required to induce STAT1 signaling and its nuclear translocation (**Table S4C**). IFN- only induced the previously identified components of the complement system, *C3*, *C1R*, *C1S*, *CFB*, and *IRF7* and *IRF9*, in the presence of replete STAT1 status (**Fig. 5D**). Moreover, most SARS-CoV-2 induced genes normalized by ruxolitinib were down-regulated in *STAT1^/^* cells and did not respond to IFN- treatment (**Fig. S10A**). These data indicated that STAT1 was indispensable for inducing these genes. In addition, *IL6* transcription was also not induced by IFN- treatment in these cells, irrespective of STAT1 status (**Fig. 5D**), so we concluded that STAT1, not STAT3, was the dominant driver of complement gene regulation. To address the concern that JAK-STAT inhibition could impair anti-viral immunity and enhance viral replication (*26*), we quantified SARS-CoV-2 viral loads in these samples by aligning raw reads to the viral genome. Ruxolitinib treatment did not alter viral loads in any of these samples (**Fig. S10B**).

### SARS-CoV-2-driven C3 activation could be normalized by CFB or JAK-STAT1 inhibition

**Fig. 6:**
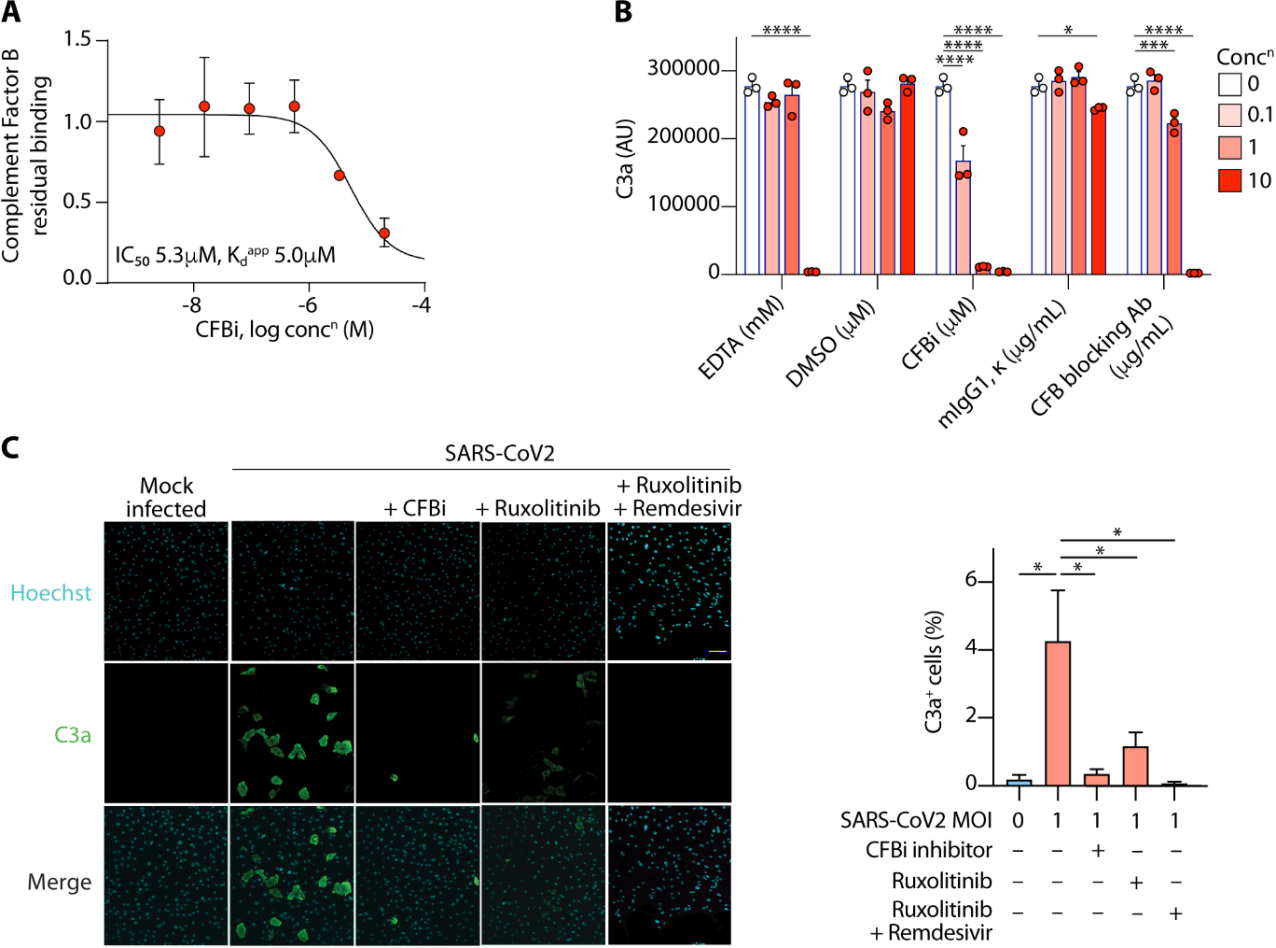
Pharmacological inhibition of key targets inhibited C3a output from SARS-CoV-2-infected respiratory epithelial cells. (**A**) chemoproteomic profiling of CFBi identified complement factor as the only target. Shown is the dose-dependent reduction of bead-binding of complement factor B from protein extracts of cells. Shown are mean and s.d. from 3 independent experiments. (**B)** C3a ELISA in plasma treated with zymosan (an alternative complement pathway activator) in the presence of increasing concentrations of EDTA (a chelator of divalent cations, which stops convertase activity), a CFB blocking antibody or isotype control, the chemical CFBi or its carrier, DMSO. Bars show mean + sem; dots represent individual experiments. (**C**) confocal images (left) and quantifications (right) showing generation of C3a in mock- or SARS-CoV-2-infected iAEC2s treated with CFBi, ruxolitinib or a combination of ruxolitinib and remdesivir. Scale bar indicates 100m. Data are from *n*=2 independent experiments; 18191 + 660 (mean + sd) cells per condition. Bars indicate mean + sd (**A**) or sem (**B-C**). *p<0.05, ***p<0.001, ***p<0.0001 by ANOVA.

We identified *CFB* as one of the most highly induced complement genes in response to SARS-CoV-2 (**Figs. 1F-H**), which suggested the potential synthesis of an inducible cell-intrinsic C3 convertase in infected cells. To test this possibility, we first investigated the ability of a novel cell-permeable inhibitor of CFB (CFBi) to reduce virus-induced C3a production in SARS-CoV-2 infected iAEC2s. This inhibitor specifically targets CFB (**Fig. 6A** and **Table S5**), rapidly diffuses into cells (**Fig. S11A**) and blocks complement activation induced by zymosan, a strong alternative complement pathway complement activator (**Fig. 6B**), without inducing cell death (**Fig. S11B**). Addition of this CFBi to cultures dramatically reduced C3a generation in response to SARS-CoV-2 infection (**Fig. 6C**), confirming that C3 processing in response to virus occurs via a cell-intrinsic C3 convertase in respiratory epithelial cells.

We also tested the ability of ruxolitinib, alone or in combination with remdesivir, to block C3a production in cells. Ruxolitinib significantly inhibited C3a generation and this effect was further inhibited by the presence of remdesivir (**Fig. 6C**), consistent with the transcriptional data (**Fig. 5C**). None of the drugs reduced SARS-CoV-2 N-protein expression but they did reduce syncytia formation (**Figs. S12A-B)**, which may indicate that complement may also influence the biology of the virus in cells. Taken together, these data confirmed that SARS-CoV-2 induced *C3* transcription via JAK-STAT1 signaling and that C3 was activated via an alternative pathway convertase that was intrinsic and inducible in infected cells. Blockade of JAK1/2STAT1 signaling, particularly in combination with an anti-viral, normalized the production of C3a from infected cells.

## DISCUSSION

**Fig. 7:**
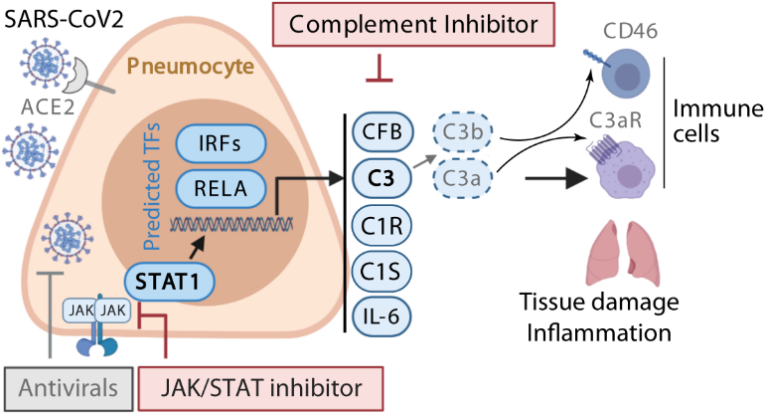
**Schematic model of SARS-CoV-2-induction of complement in respiratory epithelial cells**. SARS-CoV-2 infects respiratory epithelial cells and induces an interferon response. IFNs signal via the IFN receptor to activate STAT1 via JAK1/2. STAT1 co-operates with RELA to induce transcription of IL6 and complement genes including *C3*, *CFB*, *C1R* and *C1S*. CFB acts as an alternative pathway C3 convertase to cleave C3 intracellularly to C3a and C3b. C3a engages C3aR and C3b engages CD46 on leukocyte subsets in the lungs to drive inflammation. These events can be pharmacologically targeted with antivirals (e.g., remdesivir), JAK-STAT inhibitors (e.g., ruxolitinib) and/or cell permeable complement inhibitors, including CFBi.

Here, we showed that the induction of complement expression and C3 protein activation in airway epithelial cells is a SARS-CoV-2-driven event and not a bystander event triggered by overt cytokine production/inflammation. C3 was induced in response to SARS-CoV-2 in infected epithelial cells in a JAK/STAT-dependent manner and then processed to biologically active C3a by CFB. This could be normalized by pharmaceutical blockade of the relevant pathways (**Fig. 7**).

Complement activity is usually protective during viral infections and required to control pathogens (*27*). However, excessive activation of complement contributes to acute respiratory distress syndrome (ARDS) caused by a number of different etiologies (*28*, *29*) and is a mediator of acute lung injury driven by pandemic respiratory viruses (*30*, *31*). Since the observed gene signatures in SARS-CoV-2 were inflammatory, we conclude that C3 ligation of its receptors on tissue resident/infiltrating leukocytes during SRAS-COV2 infection is pathogenic. This may be a mechanism common to pandemic coronaviruses, since mouse models of SARS-CoV1 infection, a related virus of the same family, have indicated that *C3*, *C1r* and *Cfb* are all part of a pathogenic gene signature correlating with lethality (*32*), and that global *C3^/^* status is protective (*33*). Patients with severe COVID-19 have high circulating levels of terminal activation fragments of complement (C5a and sC5b-9) (*8*,*10*), which correlate with the clinical severity of disease (*8*). In fact, C3 hyperactivation is an independent risk factor for mortality from COVID-19 (*12*). In support of these observations, single nucleotide variants in genes encoding two complement regulators, decay accelerating factor (*CD55*) and complement factor H (*CFH*), are risk factors for morbidity and mortality from SARS-CoV-2 (*11*). Additionally, encouraging outcomes using inhibitors clinically point to a pathogenic role for complement in severe COVID-19, although such small case series should be interpreted with caution. Notably, the C3 inhibitor AMY-101 has been used in four patients with COVID-19, who recovered (*34*, *35*); the C5 activation inhibitor, eculizumab, as adjunctive treatment in 14 patients, 12 of whom recovered (*35*, *36*); another anti-C5a antibody (BDB-001) was used in 2 patients with critical COVID-19, who recovered (*37*). Moreover, avdoralimab, a human Fc-silent monoclonal antibody against C5aR1, inhibited production of inflammatory cytokines, induced by either C5a or single strand RNA virus-like stimuli, by monocytes of patients with COVID-19 (*8*). Nonetheless, the mechanism that converts the protective complement system into a harmful one during COVID-19 is currently unclear but may be rooted in the overwhelming combined local and systemic complement induced by the virus. Our findings indicated that infection of respiratory epithelial cells is a potent inducer of active complement within the lungs. This is significant because serum-derived complement was absent here, so locally produced complement could represent the major source. Corroborating sustained local activation of this system, a published clinical study recently reported the presence of both proximal (C4d) and distal complement fragments (C5b-9) in lung tissues (*38*).

Our data indicated that IFN-induced STAT1 is the dominant regulator of local complement production from respiratory epithelial cells and that the JAKi ruxolitinib neutralizes SARS-CoV-2-mediated complement activation but does not fully normalize the transcriptome. Little is known about the regulation of local complement expression; thus, these data have relevance beyond SARS-CoV-2 infection. Potential connections between complement activity and type I IFN responses during pathogen encounter is currently an unexplored field aside from a study that used full C3-deficient animals and noted suppressive effect of C3 on IFN after exposure of animal to plant virus-like nanoparticles (*39*). There is a proposed link between IFNs and complement in the context of transplant thrombotic microangiopathy (*40*). Our data suggest that the use of a JAKi to normalize all of the proximal genes induced by SARS-CoV-2 could represent a more refined approach than targeting a single complement component (e.g., C3 or C5a) with inhibitors that only work in the extracellular space. Complement activation also occurs in the intracellular space, where it performs functions critical to mounting an effective inflammatory immune response (*6*, *7*) (**Fig. 7**). Moreover, interfering with type I IFN signaling can redirect immunity to enable control of viral infection (*41*). In fact, the dual nature of type I IFNs in SARS-CoV-2induced inflammation has recently been elegantly reviewed by others (*42*). The observation that these drugs also reduced syncytia formation are intriguing since they suggest that complement may also influences the biology of the virus in cells CD46, the receptor for C3b, the other major product of C3 processing, is known to enhance syncytia formation in other viral infections (*43*), so it is possible that reducing active C3 also reduces syncytia formation.

The prediction that the NF-B pathway is also a regulator of the genes induced by SARS-CoV-2 and the failure of ruxolitinib to normalize a large subset of genes suggests that monotherapy with ruxolitinib may be insufficient for the management of COVID-19. While there are ongoing clinical trials with JAKi for treating patients with COVID-19, our analyses suggest that combining a JAKi, such as ruxolitinib, with for example anti-viral agents (e.g., remdesivir), may be a more promising therapeutic approach than monotherapy alone. Because of concerns regarding the use of JAKi in a disease with a propensity to thrombosis (*1*, *44*, *45*), combining a JAKi with a second agent may permit use of lower doses of both drugs, potentially reducing thrombotic adverse effects and reducing risks of viral replication. With regards to concerns that JAKi can increase viral replication, severe COVID-19 is characterized by hyper-inflammation and viral loads are actually low at this point, so reducing inflammation is of primary importance, thus potentially increasing viral load with a JAKi is a lesser concern than in milder or the initial stages of disease. In conclusion, transcription and activation of pathogenic complement can be normalized in epithelial cells using drugs that target JAK1, CFB or the virus itself and combinations of these may be therapeutically beneficial in treating COVID-19.

## MATERIALS AND METHODS

### Study design

The objective of this study was to investigate host-virus interactions in SARS-CoV-2 infected cells. We analyzed bulk and single cell RNA-sequencing data from the blood and lung tissues of patients with COVID-19 and compared them to healthy controls. To verify activation of complement in situ within cells, we infected iAEC2 and Calu-3 cells with SARS-CoV-2 and carried out confocal microscopy for C3a. We performed computational drug prediction to identify those capable of normalizing gene signatures induced by SARS-CoV-2. To verify our top predictions in a culture system, we infected iAEC2s with SARS-CoV-2 with and without addition of the respective cultures to the culture medium.

### Cell culture and viral infections

Human adenocarcinoma lung epithelial (Calu-3) cells (ATCC, HTB-55) were cultured in Dulbeccos Modified Eagle Medium (DMEM, GIBCO) supplemented with 10% Fetal Bovine Serum (FBS, Corning), HEPES, non-essential amino-acids, L-glutamine and 1X Antibiotic-Antimycotic solution (Gibco). The iPSC (SPC2 iPSC line, clone SPC2-ST-B2, Boston University) derived alveolar epithelial type 2 cells (iAEC2s) were differentiated as previously described and maintained as alveolospheres embedded in 3D Matrigel in CK+DCI media, as previously described (*46*). iAEC2s were passaged approximately every two weeks by dissociation into single cells via the sequential application of dispase (2mg/ml, Thermo Fisher Scientific, 17105-04) and 0.05% trypsin (Invitrogen, 25300054) and re-plated at a density of 400 cells/l of Matrigel (Corning, 356231), as previously described (*46*). All cells were maintained at 37C and 5% CO2.

SARS coronavirus 2 (SARS-CoV-2), Isolate USA-WA1/2020 (NR-52281) was obtained from Bei resources and was propagated in Vero E6 cells in DMEM supplemented with 2% FBS, 4.5 g/L D-glucose, 4 mM L-glutamine, 10 mM Non-Essential Amino Acids, 1 mM Sodium Pyruvate and 10 mM HEPES. Infectious titers of SARS-CoV-2 were determined using TCID50 method (*47*). The mock virus was prepared similarly using supernatant of Vero E6 cells. Ten thousand Calu-3 and iAECs per well were seeded in 384 well plate (PerkinElmer, 6057300) and allowed to form 80% confluent monolayer. SARS-CoV-2 virus was pre-treated with porcine trypsin (10ug/ml) for 15 min at 37 degrees. Cells were then infected or mock-infected with pre-treated virus prep at a multiplicity of infection (MOI) of 2 for Calu-3 and 1 for iAECs for 1h in culture media (final concentration of trypsin on cells was 2ug/ml). After absorption, virus inoculum was removed and replaced with fresh culture media. In the experiments with compound treatment conditions, virus inoculum was replaced with media containing a cell permeable complement factor B inhibitor (CFBi, 2 M, GlaxoSmithKline), ruxolitinib (1M, Cayman Chemical Company, cat nr. 11609) or a combination of ruxolitinib and remdesivir (250nM, Cayman Chemical Company, cat nr. 30354).

All experiments using SARS-CoV-2 were performed at the University of Michigan under Biosafety Level 3 (BSL3) protocols in compliance with containment procedures in laboratories approved for use by the University of Michigan Institutional Biosafety Committee (IBC) and Environment, Health & Safety (EHS).

### Confocal imaging and analysis

Two days post infection (p.i.), mock or SARS-CoV-2 infected cells were fixed with 4% PFA for 30 min at room temperature, permeabilized with 0.3% Triton X-100 for 15 min, and blocked with antibody buffer (1.5% BSA, 1% goat serum, and 0.0025% Tween 20). The plates were then sealed, surface decontaminated, and transferred to BSL2 for staining. To detect virally-infected cells, anti-nucleocapsid protein (anti-N) SARS-CoV-2 antibody (Sino Biological, cat nr. 40143-R019) was used as a primary antibody with an overnight staining at 4C followed by staining with secondary antibody Alexa-647 (goat anti-rabbit, Thermo Fisher, A21245). To detect activated C3, C3a antibody was used with the same staining protocol as the viral marker by using an anti-human C3a neo-epitope (Abcam, cat nr. 2991) and a secondary antibody Alexa-488 (goat anti-mouse, Thermo Fisher A21121). Hoechst-33342 pentahydrate (bis-benzimide) was used for nuclei staining (Thermo Fisher, H1398).

Plates were subjected to confocal imaging using a Thermo Fisher CellInsight CX5 High-Content Screening Platform (Calu-3) or a Yokogawa CQ1 Benchtop High-Content Analysis System (iAECs). Images were analyzed using the open-source CellProfiler (3.1.9) software with a pipeline designed to segment cell nuclei, cytoplasm, and infected regions (either syncytia or singly infected cells) given staining for Hoechst 33342 and N protein. Intensity of C3a, and N protein were measured within nuclei and cytoplasm on a per-cell level along with Pearson correlation of C3a intensities to that of N protein. Cells were considered syncytial infected if their nuclei were found present within a viral region. The distribution of N protein signal intensities in mock infected cells was calculated and cells above (mean+1.96 sd) signals were considered as infected. Visual inspection of the images confirmed the validity of this method in determining infection rate. The C3a signal intensities were then plotted as a function of infection or N protein signal intensities.

### Chemoproteomic profiling of CFBi

The human biological samples were obtained with institutional ethics approvals, and their research use was in accord with the terms of the informed consents under an IRB/EC approved protocol. To generate a bead matrix an amine-functionalized analog, of CFBi termed CFBi-F (produced by GSK), was immobilized on sepharose beads. To distinguish between proteins binding to the immobilized compound and background, a quantitative competition-based approach was applied. The test compound CFBi-F was spiked into aliquots of protein extract (mixed HEK293, K-562, placenta, HepG2 cell/tissue extract) over a range of concentrations starting at 20M in 1:6 dilutions. It then competed with the immobilized analog for binding to the target protein/s. Matrix-bound proteins were eluted, trypsinized and subsequently encoded with isobaric mass tags (TMT10) enabling relative quantification by LC-MS/MS. Only the captured target protein of the test compound - Complement Factor B was dose-dependently reduced from bead-binding, thus enabling the determination of concentrations of half-maximal binding (IC_50_). Apparent dissociation constants (K_d_^app^) were derived from the IC_50_ values by taking into account the amount of target sequestered by the affinity-matrix using the Cheng-Prusoff relationship (IC_50_/ K_d_^app^ correction factor) and sequential binding experiments. IC_50_-values for Complement Factor B competition were averaged and standard deviation calculated. Values average of the 3 independent experiments are indicated in the figure.

### Serum complement alternative pathway assay to measure activity of CFBi

The biotinylated C3a (Clone 2991, Hycult HM2074BT-B) capture antibody was diluted in MSD Diluent 100 (catalog number: R50AA-2) and was added to MSD GOLD 96-Well Streptavidin SECTOR Plate (catalog number: L15SA-1). Following 1h incubation the plate were washed 3 times in PBS with 0.05% Tween 20 and blocked with blocking solution (2% BSA in PBS + 0.05% Tween) overnight at 4C. The next day, normal human plasma was diluted to 6% in alternative pathway buffer (7.5mM HEPES, 150mM NaCl, 7mM MgCl_2_, 10mM EGTA) and activated with zymosan (1mg/ml). Small molecule Factor B inhibitor (CFBi) and factor B blocking antibody with appropriate negative (DMSO vehicle for CFBi and IgG1 isotype antibody) and positive control (EDTA) were added to wells of the 96-well U-bottom plate and incubated for 1.5 hours at 27C with shaking at 750rpm. The reaction was stopped with 5mM EDTA and 50l aliquot was transferred to the anti-C3a coated MSD plate. A C3a standard curve was constructed using purified human C3a (ComTech, A118) in concentrations ranging from 10nM to 0.00977nM. Following 1.5 hours of incubation at 27C with shaking the plates was washed in PBS/0.05% Tween 20 and the detection ruthenylated anti-C3a antibody (clone 474, made at GSK) was added. Following 3 washes, plates were developed with MSD Read buffer and electroluminescence was recorded on MSD Sector 6000 plate reader.

### Determination of cell viability using flow cytometry

CD4^+^ T cells were isolated from human whole blood drawn. For their isolation STEM CELL CD4^+^ T cell negative selection kit (#17952) was used according to manufacturers provided protocol. Cells were treated with 10M final concertation of cell permeable CFB inhibitor or equivalent volume of vehicle control (DMSO) and activated in 96 well flat bottom plates (Greiner, #655083) coated with 1g/ml anti-CD3 antibody (clone OKT, Biolegend, #317347) and 2g/ml anti-CD28 antibody (Thermo Fisher, #16-0289-85). Following 48 hours in culture, cells were transferred into 96-well V-bottom plate and analyzed for FSC and SSC properties on BD FACS Canto. Data shown is percent of viable cells in three healthy donors.

### Determination of intercellular CFBi compound concentrations

#### Sample preparation

Human CD4^+^ T cells were isolated from peripheral blood as described above. Human CD14^+^ monocytes were isolated using CD14 negative isolation kit (STEM CELL, #17858) as per manufacturers protocol. Cells were then washed twice in ice cold sterile PBS and adjusted for concertation 1x10^6^/ml in RPMI 1640 supplemented with 10% FCS. Two hundred and fifty thousand cells/well from both CD4^+^ and CD14^+^ populations were added to 48 well plates (Greiner, #677180) and 10M of CFBi or equivalent amount of DMSO was added to the wells. CD4^+^ T cells were activated with anti-CD3/antiCD28 antibodies as described above while CD14^+^ monocytes were activated with 100ng/ml LPS. Cells were harvested via centrifugation at 350*g* at 60 and 120 min post activation and supernatant removed. The pellets were snap frozen at -80C until used.

#### Standard curve preparation

Standard curves 0.1 10,000 ng/mL over 16 points (0.1, 0.2, 1, 2, 5, 10, 20, 50, 100, 200, 500, 1000, 2000, 5000 and 10000 ng/mL) were constructed in relevant matrices and these along with the samples (25 mL) were quenched with 300 mL acetonitrile containing Reserpine at 175 ng/mL as the internal standard. All samples were shaken for 20 min on a vortex mixer then centrifuged for 15 min at 1600 *g*. A 1.5 L aliquot of the resulting supernatant was injected to the mass spectrometer for analysis.

#### HPLC-mass spectrometry apparatus, conditions and data interpretation

The HPLC system was an integrated Shimadzu modular HPLC system comprising of two LC-30AD binary pumps, SIL-30ACMP autosampler, CTO-20C column oven, CBM20Alite controller (Shimadzu, Milton Keynes, Buckinghamshire, UK). The HPLC analytical column was a Kinetex Evo C18 2.5 u 50 mm 2.1mm (Phenomenex Ltd, Macclesfield, Cheshire, UK) maintained at 40C. The mobile phase solvents were water containing 0.1% formic acid and acetonitrile containing 0.1% formic acid. A gradient ran from 5% to 95% ACN + 0.1% formic acid up to 1 min held for 0.1 min and returning to the starting conditions over 0.15 min then held to 1.7 min at a flow rate of 0.8 mL/min. Mass spectrometry detection was by an API 4000 triple quadrupole instrument (AB Sciex, Warrington, Cheshire, UK) using multiple reaction monitoring (MRM). Ions were generated in positive ionization mode using an electrospray interface. The ionspray voltage was set at 4000 V and the source temperature was set at 650C. For collision dissociation, nitrogen was used as the collision gas. The MRM of the mass transitions for CFBi (m/z 487.17 to 308.10) and Reserpine (m/z 609.38 to 195.10), were used for data acquisition. Data was collected and analyzed using Analyst 1.4.2 (AB Sciex, Warrington, Cheshire, UK), for quantification, area ratios (between analyte/internal standard) were used to construct a standard line per analyte and results extrapolated from the area ratio of samples from these standard lines.

### Gene set enrichment analysis

Gene set enrichment analysis (GSEA) was performed using GSEA version 4.0.3 (*14*) with the following parameters, Permutation type = gene_set and Collapse to gene symbols = No_Collapse. All canonical pathways c2.cp.v7.1 were used throughout the paper. Upstream transcriptional regulator identification was done using Ingenuity Pathway Analysis (IPA) on genes that were differentially expressed (fold change 1.5, FDR <0.05) in NHBE or A549 cells. GSEA in **Figs. S2C-E** was performed using pre-ranked and No Collapse options.

### Bulk RNA-seq data analysis

For GSE147507, the original raw read counts were normalized to obtain transcript per million (TPM) values which were then used for plotting the expression values and performing GSEA analyses. For SRP257667, the raw fastq files were downloaded and mRNA expression levels were estimated by RSEM software (*48*) using rsem-calculate-expression with the following parameters, bowtie-n 1 bowtie-m 100 seed-length 28. The RSEM required bowtie index was created by rsem-prepare-reference on all RefSeq genes downloaded from UCSC table browser on April 2017. The differentially expressed genes were identified using edgeR package (*49*) on the original raw read counts for GSE147507 and the expected read counts from rsem for SRP257667. Fold change (FC>1.5) and FDR q-value (p<0.05) were used to identify differentially expressed genes. Viral RNA load (viral titer) was calculated by counting the fraction of all mapped sequencing reads aligned to the corresponding viral genome (RSV: M11486.1, IAV: NC_002023.1, SARS-CoV-2: NC_045512.2), indicated as parts per million reads of library size, P.P.M. (*50*) in each sample.

### ChIP-seq data analysis

H3K27Ac ChIP-seq in A549 cells (ENCFF137KNW), H3K27Ac ChIP-seq in primary lung cells (ENCFF055YQO, ENCFF677KZQ) and STAT1 ChIP-seq in HeLa cells (ENCFF000XLN) were obtained from ENCODE. RELA ChIP-seq in FaDu cells was from GSE132018. In all cases, the pre-processed and author-provided peak files (e.g., ENCFF565WST, ENCFF002CTG) were obtained and the nearest transcription start sites (TSS) and corresponding genes were identified by HOMER annotatePeaks program. The overlap between these genes and SARS-CoV-2 induced/repressed genes or all human genes were then assessed to determine enrichment. ChIP-seq tracks and heatmaps were visualized using IGV browser (Broad Institute) and deepTools (*51*), respectively.

### Statistical analysis and data visualization

Analyses were performed using GraphPad PRISM 8 (La Jolla, CA, USA) and Data Graph v4.5. All the individual data points are presented and compared using one-way ANOVA or Fisher exact test, as appropriate. *P* values <0.05 are denoted as statistically significant throughout. The heatmaps were drawn using Morpheus software (Broad Institute). The schematic in **Fig. 7** was created with Biorender.com.

### Drug prediction

Raw read counts for A549 and NHBE cells comparing the SARS-CoV-2 infected cells with controls were obtained from GSE147507 and normalized to obtain TPM values (see **Table S1A**). Drugs with provided down-regulated target genes (between 10 to 1000) were obtained from DSigDB v1.0 (*22*). For ruxolitinib, the lists of all up and down-regulated genes (p-value<0.05) were obtained by comparing MCF-7 cells treated with ruxolitinib or vehicle control (data from GSE131300) using DESeq2 (*52*) (See **Table S4D**). For baricitinib, the list of all up and down-regulated genes (p-value<0.0005) were obtained by comparing systemic baricitinib treatment versus control at 12 weeks (data from GSM1508095) using GEO2R (*53*) (See **Table S4D**). The gene set enrichment analysis (GSEA) was performed using GSEA version 4.0.3 with the following parameters, Permutation type = gene_set, Collapse to gene symbols = No_Collapse, Min Size =10 and Max Size =1000. A549 and NHBE samples were treated as Expression datasets and the DSigDB data was treated as Gene sets database. All the rest of the parameters were kept as default. The data with FDR q-value <0.25 was reported (See **Table S4A**).

### Single Cell RNA sequencing data analysis (BALF)

The pre-processed h5 matrix files for six COVID-19 patient bronchoalveolar-lavage (BAL) samples and eight uninfected control lung biopsies were obtained from GSE145926 and GSE122960, respectively. Read mapping and basic filtering were performed with the Cell Ranger pipeline by the original authors. We further processed the samples using Seurat (version 3) as following: Only genes found to be expressed in more than 3 cells were retained. Cells with >10% of their unique molecular identifiers (UMIs) mapping to mitochondrial genes or cells with <300 features were discarded to eliminate low quality cells or nuclei. This yielded a total of 89133 cells across 14 samples. The filtered count matrices were then normalized by total UMI counts, multiplied by 10,000 and transformed to natural log space. The top 2000 variable features were determined based on the variance stabilizing transformation function (FindVariableFeatures) by Seurat with default parameters. All samples were integrated using canonical correlation analysis (CCA) function with default parameters. Variants arising from library size and percentage of mitochondrial genes were regressed out by the ScaleData function in Seurat. Principal component analysis (PCA) was performed and the top 30 Principal components (PCs) were included in a Uniform Manifold Approximation and Projection (UMAP) dimensionality reduction. Clusters were identified on a shared nearest neighbor (SNN) modularity graph using the top 30 PCs and the original Louvain algorithm. Cluster annotations were based on canonical marker genes. Gene list scores were calculated by AddModuleScore function in Seurat (*54*). Statistical differences of marker expressions and scores were assessed by Wilcoxon test.

### Single Cell RNA sequencing data analysis (PBMC)

The pre-processed serialized R objects for (*n*=6) COVID-19 patient peripheral blood mononuclear cell (PBMC) samples and (*n*=6) healthy control PBMC samples were obtained from GSE150728 (*55*). Read mapping and basic filtering were performed with the Cell Ranger pipeline (10x genomics) by the original authors. The exonic count matrices were further processed by Seurat (version 3) as follows: Only genes found to be expressed in more than 10 cells were retained. The QC steps for filtering the samples were performed as described (*55*). Briefly, Cells with 1,000-15,000 UMIs and < 20% of reads from mitochondrial genes were retained. Cells with > 20% of reads mapped to RNA18S5 or RNA28S5, and/or expressed more than 75 genes per 100 UMIs were excluded. SCTransform function was invoked to normalize the dataset and to identify variable genes as previously described (*55*). Principal component analysis (PCA) was performed and the top 50 principal components (PCs) were included in a Uniform Manifold Approximation and Projection (UMAP) dimensionality reduction. Clusters were identified on a shared nearest neighbor (SNN) modularity graph using the top 50 PCs and the original Louvain algorithm. Cluster annotations were based on canonical marker genes. Gene list module scores were calculated by the AddModuleScore function in Seurat with a control gene set size of 100.

## Supplementary Materials

immunology.sciencemag.org/cgi/content/full/6/58/eabg0833/DC1 Fig. S1. SARS-CoV-2 infection activates complement transcription in lung epithelial cells. Fig. S2. SARS-CoV-2 infection activates complement transcription in human lung organoids and primary human bronchial epithelial cells. Fig. S3. C3 protein is induced by SARS-CoV-2 infection of respiratory epithelial cells. Fig. S4. Complement components are expressed in lymphoid, myeloid and epithelial cells. Fig. S5. SARS-CoV2 infection activates complement transcription in bronchoalveolar lavage fluid (BALF) cells. Fig. S6. SARS-CoV2 viral load correlates with C3 mRNA expression in lung tissues of patients with COVID-19. Fig. S7. BALF cells from COVID-19 patients have a CD46 activated signature. Fig. S8. SARS-CoV2 infection minimally affects complement pathways in PBMC across different cell types. Fig. S9. STAT1 and RELA bind SARS-CoV2-induced genes. Fig. S10. STAT1 dependence of SARS-CoV2-induced genes. Fig. S11. Data showing activity of cell-permeable complement factor B inhibitor (CFBi). Fig. S12. Percentage N-protein+ and syncytial cells in SARs-CoV2 infected iAECs treated with chemical agents. Table S1. Normalized expression of all transcripts in GSE147507 and SRP257667 and GSEA outputs used in Figure 1 Table S2. List of CD46, C3aR and Interferon alpha/beta target genes curated experimentally for CD46 and from literature for the rest. Table S3. Differentially expressed genes in SARS-CoV-2-infected cells. Table S4. GSEA-based drug prediction. Table S5. Mass spectrometry data for profiling of CFB inhibitor. Table S6. Data sources used in this study. Table S7. Table of raw data.
